# Telomerase deficiency reflects age-associated changes in CD4+ T cells

**DOI:** 10.1186/s12979-022-00273-0

**Published:** 2022-03-23

**Authors:** Diana M. Matthe, Oana-Maria Thoma, Tobias Sperka, Markus F. Neurath, Maximilian J. Waldner

**Affiliations:** 1 Department of Medicine 1, Friedrich-Alexander-Universität Erlangen-Nürnberg, Universitätsklinikum Erlangen, Ulmenweg 18, 91054 Erlangen, Germany; 2 Erlangen Graduate School of Advanced Optical Technologies (SAOT), Friedrich-Alexander-Universität Erlangen-Nürnberg, Erlangen, Germany; 3 Leibniz Institute on Aging, Fritz Lipmann Institute, Jena, Germany

**Keywords:** Telomere shortening, Telomerase, CD4-positive T-lymphocytes, Th1 cells, Aging

## Abstract

**Background:**

Amongst other systemic changes, aging leads to an immune dysfunction. On the molecular level, a hallmark of aging is telomere shortening. The functional relevance of telomerase, an enzyme capable of elongating telomeres in T cells upon antigen stimulation, is not fully understood. Studying the impact of telomere shortening on CD4+ T cells and especially Th1 effector function can provide a better understanding on immune dysfunctions in elderly.

**Results:**

We investigated T cell numbers and differentiation in telomerase-deficient (mTerc−/−) mice under steady-state conditions and the functional role of telomerase in CD4+ T cells using in vitro stimulation and Th1 polarization protocols by comparing T cells from mTerc−/− and control mice. We report reduced relative CD4+ T cell numbers in blood and secondary lymphoid organs and a relative decline in the naïve T cell population in thymus, blood and spleen of mTerc−/− mice compared to control mice. Importantly, after in vitro polarization, mTerc−/− G3 CD4+ T cells showed higher numbers of IFNγ-producing cells and reduced expression of CD28. Notably, telomerase-deficient T cells were more susceptible to inhibition of Th1 polarization by IL-6 in vitro. These results demonstrate that telomerase deficiency recapitulates several changes of CD4+ T cells seen in aged humans regarding the naïve T cell population, expression of CD28 and cytokine production.

**Conclusion:**

Our data suggest that telomere shortening could play a key role in the aging of T cell immunity, with clinical implications for immune diseases and tumor development and that mTerc−/− mice are a suitable model to study aging-related defects of adaptive immunity.

**Supplementary Information:**

The online version contains supplementary material available at 10.1186/s12979-022-00273-0.

## Background

Aging is a universal process known across species and is characterized by a progressive decline in physiological function of various organ systems [1, 2]. Amongst others, aging leads to alterations within the immune system, which are globally termed ‘immunosenescence’ [3]. Key features of immunosenescence comprise a reduced capacity to respond to new antigens, as well as insufficient protection from malignancies and autoimmune diseases in elderly [3, 4]. Within the adaptive immune system, a relative reduction in B and T cell progenitors and naïve B and T cells have been reported amongst other changes [5]. Furthermore, a typical age-associated feature found in both CD4+ and CD8+ T cells is the loss of the costimulatory molecule CD28 [3, 6]. Overall, these changes result in a decreased functional responsiveness of the aged immune system to new antigens.

Importantly, the accumulation of genetic damage with time, by exogenous or endogenous factors is widely considered a central feature and cause of aging [2]. Specific repetitive nucleotide sequences of TTAGGG at the end of linear chromosomes, called telomeres, are particularly affected by aging, as they progressively shorten with each cell division [2, 7, 8]. Consequently, most tissues undergo telomere shortening with age [9]. When telomere attrition eventually reaches a critical point – the so-called Hayflick limit –, cells enter a state of irreversible cell cycle arrest, termed replicative senescence, or alternatively go into apoptosis to avoid loss of coding DNA sequences [7, 10–12]. Telomere erosion can be counteracted by the ribonucleoprotein complex telomerase consisting of the telomerase reverse transcriptase (TERT) and the telomerase RNA component (TERC), which is able to de novo synthesize telomeric repeats onto the chromosome [13]. Although telomerase activity is highly regulated and usually not present in somatic cells, numerous studies have shown that telomerase is transiently upregulated upon antigen contact in T cells in response to T cell receptor (TCR) activation and CD28 costimulation, presumably to maintain telomere length during clonal expansion [14–17]. This ability to induce telomerase is progressively lost again with repeated stimulations and increasing degree of differentiation [18, 19].

Telomeres and telomerase seem to play a critical role in the function of the immune system. Telomere length and telomerase activity have been identified as biomarkers of ‘high-performing’ centenarians [20]. In addition, reduced telomere activity or telomere shortening in CD4+ T cells and/or leukocytes have been associated with age-related diseases like cardiovascular disease and immune-mediated diseases such as rheumatoid arthritis, type I diabetes mellitus and systemic lupus erythematosus [21–23]. Furthermore, an inherited defect in components of telomerase and/or the telomere maintenance complex in humans leads to a tendency towards reduced lymphocyte counts and a susceptibility to opportunistic infections associated with T cell immunodeficiency [1, 24, 25].

These observations highlight the crucial role of telomerase for T cell function. However, while many associations have been found between telomere length and T cell immune competency as mentioned above, the direct impact of telomerase deficiency on the function of CD4+ T cells has not been investigated so far.

In the current study, we characterized mice lacking the RNA component of telomerase (mTerc). We present evidence that the immunological phenotype of mTerc−/− mice in many ways resembles age-associated changes of the adaptive immune system both under steady-state conditions as well as after in vitro polarization. More precisely, CD4+ T cells from telomerase-deficient mice showed a reduced naïve T cell pool under steady-state conditions, as well as decreased relative numbers of CD28+ cells and an increased number of cells producing the Th1-associated cytokine IFNγ after in vitro polarization, and their differentiation was more effectively inhibited by the presence of IL-6.

## Results

### Telomerase deficiency leads to reduced relative numbers of B and T lymphocytes in blood and secondary lymphoid organs

**Fig. 1:**
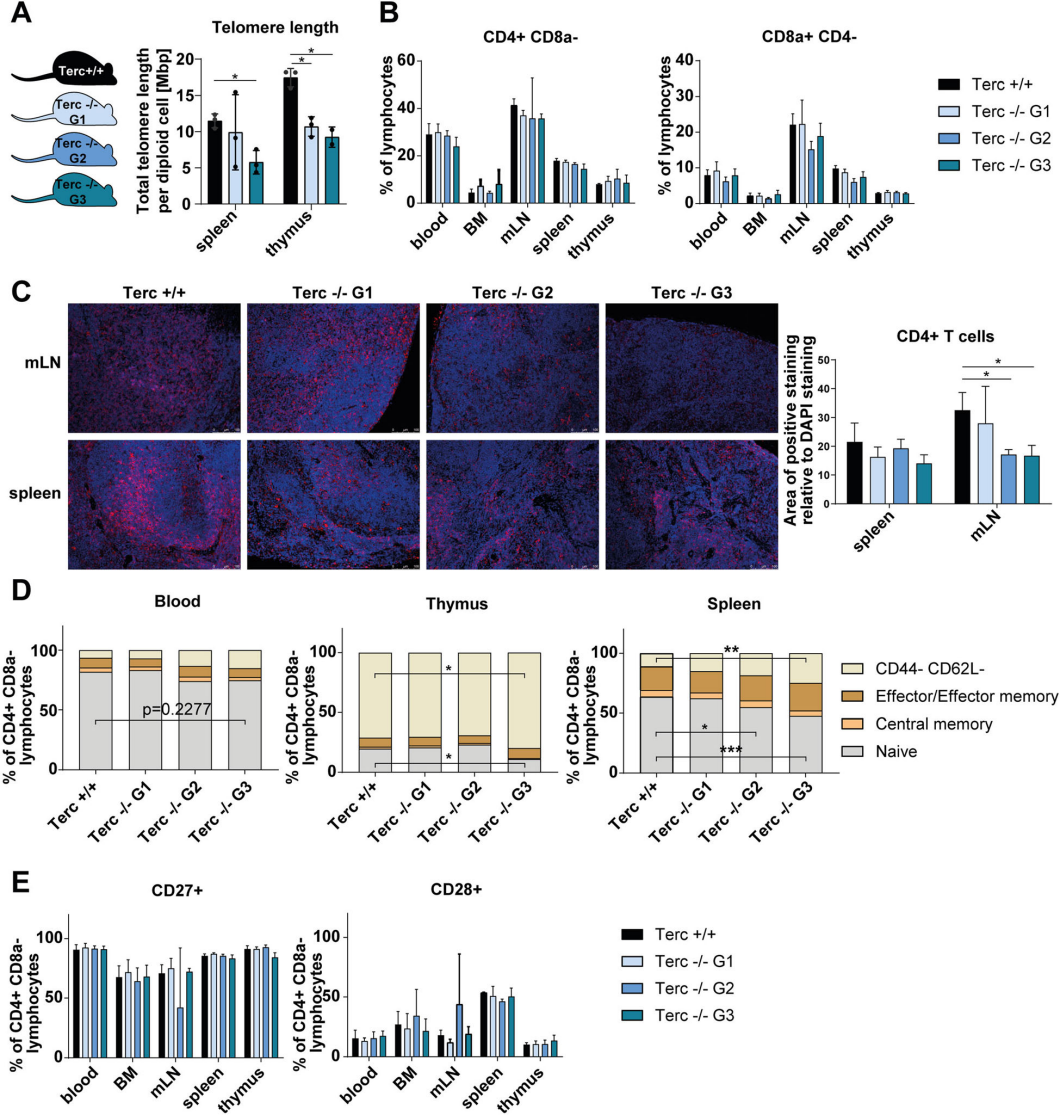
Characterization of CD4+ T cells from telomerase-deficient mice under steady state conditions. **A** Schematic representation of the different generations of mTerc−/− mice and validation of the telomere loss. Generation 2 (G2) animals were obtained by crossing mTerc−/− G1 animals, G3 animals are offspring of mTerc−/− G2 mice. Telomere length was quantified by a qPCR-based method in Terc+/+, Terc−/− G1 and Terc−/− G3 mice (*n* = 3 per group, except thymus of Terc−/− G3: *n* = 2). **B** Flow cytometric analysis of CD4+ and CD8a + cells from blood and lymphoid organs of mTerc−/− mice (*n* = 3) under steady-state conditions. **C** Immunohistochemical stainings of CD4+ T cells (red) in mesenteric lymph node (mLNs, *n* = 3 except Terc −/− G2: *n* = 2) and spleen (*n* = 3) of mTerc−/− mice. Nuclei were counterstained with DAPI (blue). Quantification was done with ImageJ software by calculation of the area of positive CD4 staining relative to the area of DAPI staining from non-overlapping pictures (≥ 5 pictures per mLN and ≥ 7 pictures per spleen). **D** Composition of the CD4+ T cell pool with regards to memory populations. Cells were defined as naïve (CD44- CD62L+), central memory (CD44+ CD62L+) and effector/effector memory (CD44+ CD62L-). **E** Relative numbers of CD4+ T cells expressing costimulatory molecules CD28 and CD27 in lymphoid organs from telomerase-deficient mice. If not otherwise indicated, the experiment was performed with *n* = 3 mice from each generation. Graphs show the mean ± SD, * adjusted *p* ≤ 0.05, ** adjusted *p* ≤ 0.01, *** adjusted *p* ≤ 0.001

Telomerase-deficient (mTerc−/−) mice with a C57BL/6 J background were bred in successive generations (G1, G2, G3) in our facility. The mice have initially been generated by Blasco et al., 1997 and have been reported to progressively lose telomere length with each generation in mouse embryonic fibroblasts and bone marrow cells [26–28]. Similarly, our measurements of cells from thymus and spleen showed a significant progressive loss in telomere length in telomerase-deficient mice (*p* values for mTerc−/− G3: 0.0444 in spleen, 0.0116 in thymus, see Fig. 1A).

To assess whether telomerase deficiency had any effect on cell numbers in lymphoid organs, we counted total cells in blood, bone marrow (BM), mesenteric lymph nodes (mLNs), spleen and thymus and found a consistent tendency towards decreased total cell counts, especially in thymus (*p* = 0.0711 for mTerc−/− G2), of mTerc−/− mice compared to controls (see Supplementary Fig. 1A). We investigated this further by examining the cell composition in these organs in more detail. We found a relative decrease of CD4+ T cells among lymphocytes in mLNs, spleens and blood of telomerase-deficient mice of generations G1-G3 (see Fig. 1B). Even though the difference is not statistically significant, the trend was clear across several generations and a stronger phenotype was observed in later generations (G3) of mTerc−/− mice. This finding could be confirmed in immunohistochemical stainings and quantification of the stained area for CD4+ (see Fig. 1C; *p* values: 0.0487 for mTerc−/− G2, 0.0425 for mTerc−/− G3 in mLN). A similar tendency was observed for CD8a + T cells and B cells (*p* = 0.0422 in spleen), which was mirrored by a relative increase of NK cells in the spleen of mTerc−/− G1 and G2 mice (see Fig. 1B and Supplementary Fig. 1B). In the analysis of T helper cell subsets, we could not identify any specific phenotype of mTerc−/− CD4+ T cells across generations (see Supplementary Fig. 1C).

### mTerc−/− mice show reduced relative numbers of naïve CD4+ T cells under steady-state conditions

We further assessed functional aspects of CD4+ T cells in mTerc−/− mice by flow cytometry. The relative numbers of naïve, central memory and effector/effector memory T cells were determined in flow cytometry by combined analysis of the activation marker CD44 and the adhesion molecule CD62L [29]. A striking relative reduction of naïve CD4+ T cells was observed especially in the spleen, but also in the blood and in thymus of G3 telomerase-deficient mice. Notably, later generations of mTerc−/− mice were affected more by this phenotype in the spleen (see Fig. 1D; *p* values: 0.0178 for mTerc−/− G3 in thymus; 0.0448 for mTerc−/− G2 and 0.0002 for mTerc−/− G3 in spleen). We could not detect this phenotype in BM or thymus (see Supplementary Fig. 2A).

Furthermore, we analysed the expression of costimulatory molecules CD27 and CD28, which are generally required for the activation of naïve CD4+ T cells by antigens via the TCR [30]. Under steady-state conditions, no specific differences in the expression of these costimulatory molecules were observed among various generations of mTerc−/− mice (see Fig. 1E).

Telomerase deficiency might lead to critically short telomeres [9, 10]. As mentioned above, this can result in a cell cycle arrest via activation of DNA damage pathways that activate cell cycle inhibitors like p21 or p16 [11]. In our studies, this did not seem to be the case in CD4+ T cells of mTerc−/− mice, as proliferation rates were not impaired under steady-state conditions (see Supplementary Fig. 2B).

Apart from cellular growth arrest, critically short telomeres might, via activation of the DNA damage pathway and p53, also lead to the induction of apoptosis [11]. Indeed, combined flow cytometric analysis of Annexin V and propidium iodide showed reduced relative numbers of live CD4+ T cells accompanied by an increase in late apoptotic CD4+ T cells in bone marrow (BM), thymus and spleen of mTerc−/− G3 mice (see Supplementary Fig. 2C). Just like the observations presented above, this was found to be more severe in successive generations (G1 to G3) of mTerc−/− mice.

### mTerc−/− CD4+ T cells show functional changes after in vitro polarization

**Fig. 2:**
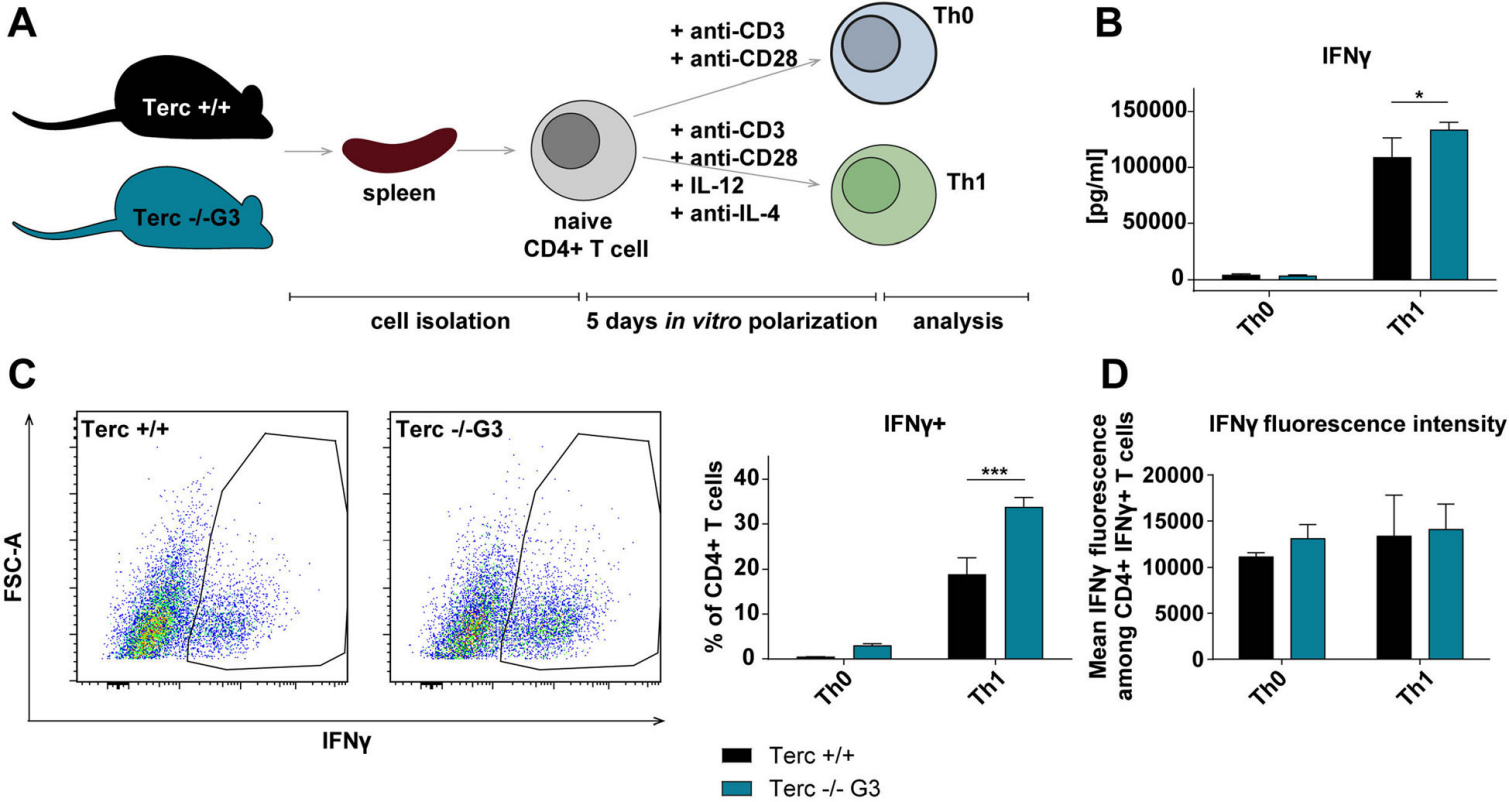
mTerc−/− CD4+ T cells show functional differences after in vitro polarization. **A** Schematic representation of the experimental setup of in vitro polarization. CD4+ T cells were isolated and stimulated in vitro for five days with anti-CD3 and anti-CD28 only (Th0) or with anti-CD3, anti-CD28, IL-12 and anti-IL-4 (Th1). On the day of analysis, the cells were treated with eBioscience™ Cell Stimulation Cocktail (plus protein transport inhibitors) (500X) (Invitrogen) for 3.5 h before analysis. **B** Quantification of IFNγ in the medium by ELISA. **C** and **D** Flow cytometric analysis of IFNγ-producing CD4+ T cells. Graphs show the mean ± SD, * adjusted *p* ≤ 0.05, ** adjusted *p* ≤ 0.01, *** adjusted *p* ≤ 0.001

Apart from steady-state conditions, we wanted to elucidate the functionality of telomerase-deficient CD4+ T cells upon stimulation. For this, we isolated naïve (CD25-) CD4+ T cells from mTerc−/− mice and stimulated with anti-CD3 and anti-CD28 (Th0) only, or treated with anti-IL-4 and IL-12 in addition to anti-CD3 and anti-CD28 for five days in vitro to induce polarization into Th1 (see Fig. 2A). Since under steady-state conditions, the later generations (G3) of mTerc−/− mice showed the strongest phenotype, we continued our in vitro analyses only with those mice.

Importantly, in vitro polarization of mTerc−/− G3 CD4+ T cells lead to a significantly higher number of cells producing the Th1-associated cytokine IFNγ (*p* = 0.000163) and higher levels of IFNγ in the medium of these cells (*p* = 0.0240) (see Fig. 2B and C). This difference became apparent only towards the end of the culture at day five (see Supplementary Fig. 3A). Remarkably, each cell did not seem to produce more IFNγ, since the mean fluorescence intensity of IFNγ+ cells stayed the same. Rather, the number of cells secreting IFNγ was higher among the mTerc−/− G3 CD4+ T cells (see Fig. 2D).

**Fig. 3:**
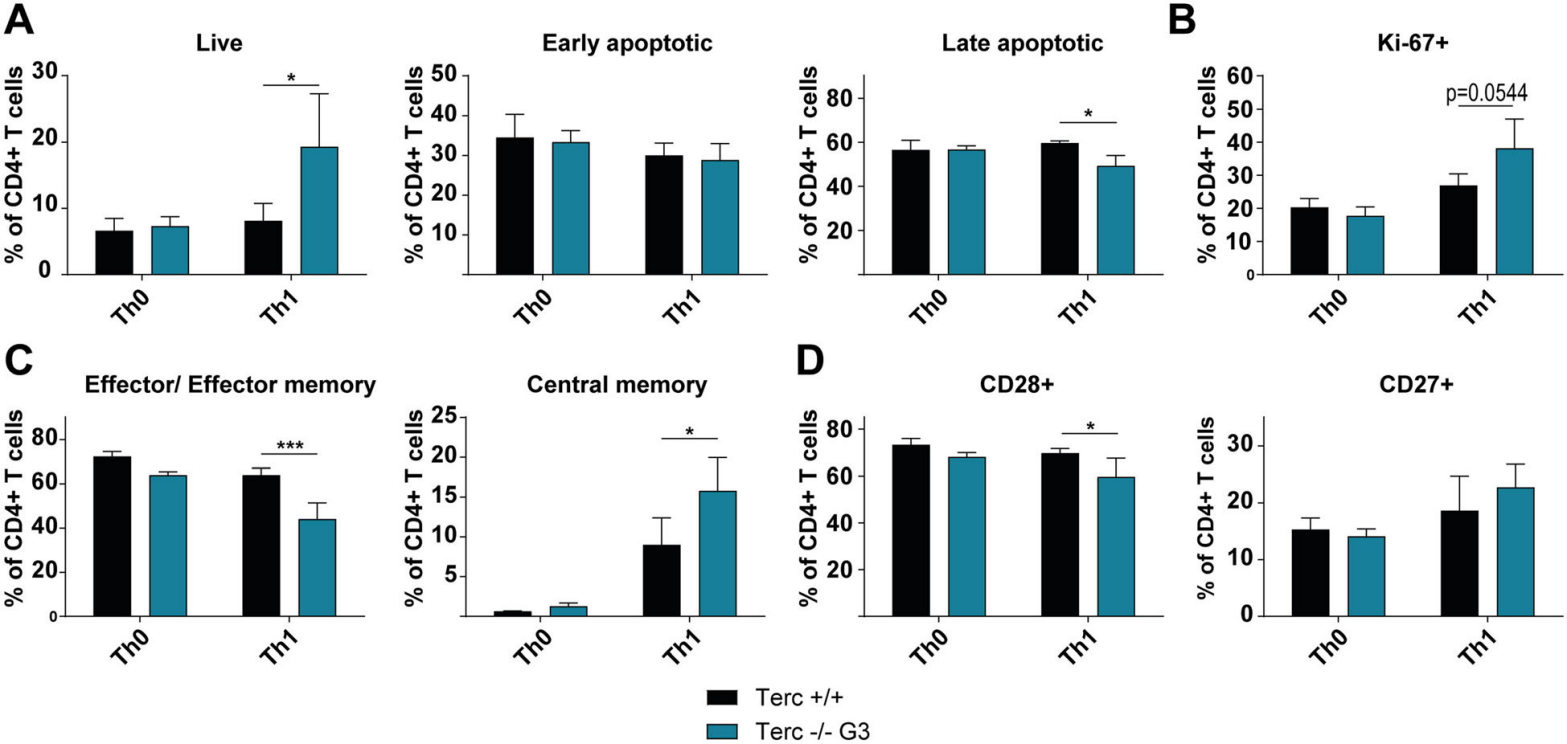
mTerc−/− CD4+ T cells show functional differences after in vitro polarization. **A** and **(B** Flow cytometric quantification of proliferating (Ki-67+), live (Annexin V- PI-), early apoptotic (Annexin V+ PI-) and late apoptotic (Annexin V+ PI+) cells. **C** Functional analysis of memory populations (central memory: CD44+ CD62L+; effector/ effector memory: CD44+ CD62L-) after in vitro polarization measured by flow cytometry. **D** Expression of costimulatory molecules CD28 and CD27 among in vitro polarized CD4+ T cells as determined by flow cytometry. All graphs show the mean ± SD, * adjusted *p* ≤ 0.05, ** adjusted *p* ≤ 0.01, *** adjusted *p* ≤ 0.001

Further, our data indicate that five days of antigenic stimulation do not suffice for telomerase-deficient G3 CD4+ T cells to go into cell cycle arrest or apoptosis, since proliferation was not impaired and apoptosis rates were not elevated. In contrast, Th1-induced cells had even higher relative numbers of proliferating and live cells (*p* values: 0.0544 and 0.0266 respectively), while apoptosis rates were significantly decreased compared to controls (*p* = 0.0100) (see Fig. 3A and B). Correspondingly, cell counts of mTerc−/− G2 were significantly higher on day 5 of in vitro polarization (see Supplementary Fig. 3B and C)*.*

These data indicate that telomerase-deficient CD4+ T cells have a preference for differentiating into IFNγ-secreting Th1 cells compared to controls.

Apart from cytokine production, other functional aspects of in vitro stimulated mTerc−/− G3 CD4+ T cells were investigated. mTerc−/− G3 CD4+ T cells tended to shift from effector/ effector memory (CD44+ CD62L-, *p* = 0.0008) towards increased central memory (CD44+ CD62L+, *p* = 0.0301) T cells (see Fig. 3C). Furthermore, the relative numbers of cells expressing the costimulatory molecule CD28 were reduced among the Th1-polarized mTerc−/− G3 CD4+ T cells (*p* = 0.0464), while no specific and reproducible differences were found in the expression of CD27 (see Fig. 3D).

These findings suggest that telomerase deficiency changes the functional properties of stimulated CD4+ T cells.

### Telomerase-deficient CD4+ T cells are more susceptible to IL-6 mediated inhibition of Th1 differentiation

As presented above, mTerc−/− G3 CD4+ T cells preferentially differentiated into IFNγ-producing Th1 cells when polarized under isolated conditions in vitro. We then wanted to further assess whether age-associated cytokines could influence Th1 cell differentiation.

As one candidate cytokine, we investigated IL-6, since it is amongst others one central component of the senescence-associated secretory phenotype (SASP), and was found to be increased in aging [31]. Furthermore, IL-6 has been reported to inhibit Th1 differentiation in aged mice [32, 33].

**Fig. 4:**
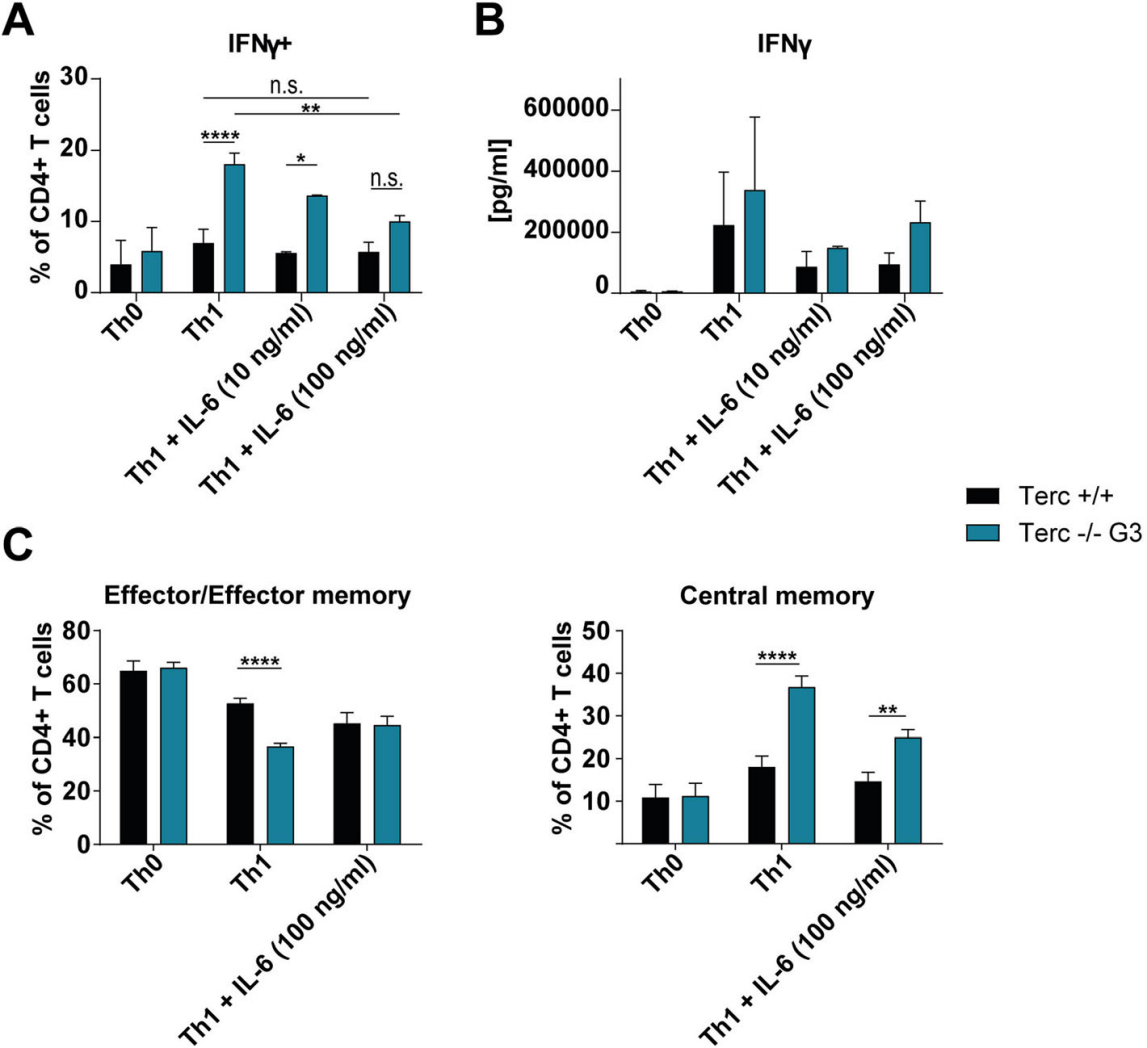
mTerc−/− CD4+ T cells are more susceptible to IL-6-mediated inhibition of Th1 differentiation. CD4+ T cells were isolated from mTerc−/− mice as before and stimulated in vitro for five days with anti-CD3 and anti-CD28 only (Th0) or with anti-CD3, anti-CD28, IL-12 and anti-IL-4 (Th1). In addition, IL-6 was added to the Th1-inducing mixture in the indicated concentrations. **A** Flow cytometric analysis of IFNγ-producing cells after in vitro polarization under IL-6 treatment. The figure depicts pooled data from two independent experiments. **B** Quantification of IFNγ in the medium after in vitro polarization of mTerc−/− CD4+ T cells. The figure shows pooled data from two independent experiments. **C** Memory populations after in vitro polarization as determined by flow cytometry (effector/ effector memory: CD44+ CD62L-; central memory: CD44+ CD62L+). Data from one measurement is shown. All graphs show the mean ± SD, * adjusted *p* ≤ 0.05, ** adjusted *p* ≤ 0.01, *** adjusted *p* ≤ 0.001, **** adjusted *p* ≤ 0.0001

When we treated telomerase-deficient CD4+ T cells with IL-6 during in vitro polarization, it mitigated the increased relative numbers of IFNγ+ among mTerc−/− G3 CD4+ T cells in a dose-dependent manner (see Fig. 4A and B). Notably, telomerase-deficient CD4+ T cells were affected to a greater extent by this inhibition, as IL-6 decreased the percentage of IFNγ+ cells only by a mean of 25% in mTerc +/+ CD4+ T cells, while it was reduced by 44% in telomerase-deficient (mTerc −/− G3) cells (pooled data from two independent experiments, *p* = 0.001269 for untreated vs. treated with 100 ng/ml IL-6 mTerc−/− G3).

These data demonstrate a specific susceptibility of telomerase-deficient cells to IL-6-mediated inhibition of Th1 differentiation, which could explain the unaltered numbers of Th1 cells under steady-state conditions in vivo*.*

We further investigated whether IL-6 also impacts other features we found in in vitro polarized mTerc−/− G3 CD4+ T cells before. Interestingly, we found that the observed shift of telomerase-deficient cells towards an increased central memory and a reduced effector/effector memory was ameliorated by IL-6 as well (see Fig. 4C; *p* values for Terc+/+ vs. mTerc−/− G3: effector/effector memory < 0.0001 in untreated and 0.9899 in IL-6-treated condition; central memory < 0.0001 in untreated and 0.0010 in IL-6-treated condition).

These data suggest that the presence of IL-6 during activation of CD4+ T cells inhibits the differentiation of Th1 cells and memory subsets specifically in aged organisms. This could have an especially high relevance given the higher serum levels of IL-6 found in elderly humans [34].

## Discussion

Aging is known to lead to a dysfunction of the immune system [4]. Telomere shortening, a hallmark of aging, has been associated with many age-related diseases [9]. Here we provide evidence that telomerase deficiency in CD4+ T cells leads to a phenotype which in many ways resembles age-associated changes in the immune system.

We observed reduced frequencies of CD4+ T cells, T cells and B cells in the blood and lymphoid organs of mTerc−/− mice. Correspondingly, decreased T cell numbers and B cell numbers have been reported in human aging as well and are considered an important contribution to age-related immune defects [35, 36].

Furthermore, we identified a relative decline of naïve CD4+ T cells in blood, spleen and thymus of mTerc−/− mice, which also correlates well with age-associated changes found within the human immune system [37, 38]. The decrease in naïve T cells with aging is assumed to be caused by a combination of the reduction in naïve T cells exiting the thymus due to thymic involution, and insufficient homeostatic maintenance of naïve T cells in the periphery [39]. However, we could not find histological signs of thymic involution in tissue sections (data not shown), and we did not observe any reduction in proliferation rates in lymphoid organs of mTerc−/− mice (see Supplementary Fig. 2B), indicating that these mice also do not have a defect in homeostatic proliferation. Similarly, Lee et al. 1998 did not report histological differences of the thymus regarding organ size, architecture or cellularity up to mTerc−/− G6 mice [27]. Furthermore, Lee et al. 1998 found the renewal of hematopoietic stem cells from the bone marrow to be markedly reduced in G6 mTerc−/− mice, while it was not yet significantly compromised in G1 or G3 mTerc−/− mice [27]. The question therefore arises what leads to the relative decline of naïve cells in G3 mTerc−/− mice. Strikingly, patients carrying an inherited mutation in telomerase were also reported to have reduced naïve CD4+ cells among peripheral cells, and this correlated with a decreased thymic output and increased apoptosis rates in the thymus of these patients, suggesting that telomerase-deficient human naïve T cells are more prone to apoptosis in the thymus [24]. Correspondingly, we observed that apoptosis rates were also significantly increased in mTerc−/− G3 mice in bone marrow and thymus. Based on this, we hypothesize that telomerase deficiency results in a susceptibility to apoptosis that affects the early developmental steps of T cells in lymphoid organs.

CD4+ T lymphocytes are capable of upregulating telomerase upon antigen stimulation, but lose telomerase activity with repetitive stimulations, which is especially seen in chronic diseases with excessive antigenic activation, such as rheumatoid arthritis, psoriasis or HIV [19].

We were able to demonstrate that a lack of telomerase (G3) during in vitro polarization of CD4+ T cells leads to a higher percentage of IFNγ-producing cells and thereby a higher amount of secreted IFNγ in the medium. This can be attributed to a higher relative amount of proliferating and live cells, as well as decreased frequencies of apoptotic cells among Th1-polarized mTerc−/− G3 CD4+ T cells.

Previous studies have reported both decreased and increased IFNγ production in aged organisms. While this seems controversial at first, different methods of analysis explain the discrepancy: When whole blood from aged individuals was obtained and stimulated in vitro, reduced amounts of IFNγ were measured in the samples from elderly subjects [40, 41]. In contrast, when specifically CD4+ T cells were isolated and stimulated in vitro, increased IFNγ levels were measured in the samples from aged humans and aged mice [42–44].

We therefore conclude that additional factors present in the blood or tissues of aged subjects could impair the otherwise increased Th1 differentiation of aged and/or telomerase-deficient CD4+ T cells that was observed under isolated conditions.

With IL-6, we identified a key element affecting IFNγ production in mTerc−/− G3 CD4+ T cells. IL-6 is known as one cytokine comprising the so-called senescence-associated secretory phenotype [31, 45]. Previous studies reported that levels of IL-6, among other inflammatory mediators, increase with age in a phenomenon termed inflamm-aging [4, 46]. Furthermore, IL-6 was shown to play detrimental roles in the aging immune system, amongst others by inhibiting the Th1 differentiation in aged mice [33, 34].

We could show that the presence of IL-6 during differentiation of CD4+ T cells strongly and almost selectively impairs the IFNγ production of mTerc−/− G3 CD4+ T cells, but not of mTerc +/+ CD4+ T cells. This indicates that telomerase-deficient CD4+ T cells are uniquely susceptible to IL-6-mediated inhibition of Th1 differentiation. Based on this, we propose that this sensitivity of telomerase-deficient CD4+ T to IL-6 in their environment is sufficient to effectively ameliorate the otherwise preferred Th1 differentiation of mTerc−/− CD4+ T cells. In contrast, telomerase-proficient CD4+ T cells seem to be less affected by IL-6 even at higher concentrations of IL-6.

Tsukamoto et al. (2015) discovered that in aged CD4+ T cells, the IL-6 mediated inhibition of Th1 polarization is mediated by IL-6 inducing the transcription of IL-4, thereby driving the differentiation to Th2 rather than Th1 [33]. In a preliminary analysis, we found that this is also the case for mTerc−/− CD4+ T cells, which expressed higher amounts of IL-4 after IL-6 treatment, and this increase was stronger than in mTerc +/+ controls (data not shown).

Interestingly, IL-6 did not only normalize the proportion of IFNγ-producing cells in telomerase-deficient CD4+ T cells. It was also able to partly reverse the phenotype of mTerc−/− G3 CD4+ T cells after in vitro polarization regarding the relative distribution of central memory and effector/effector memory populations, while not affecting memory subsets in controls. While mTerc−/− G3 CD4+ T cells seemed to have an intrinsic preference towards becoming central memory rather than effector/effector memory cells during in vitro differentiation, this preference was impaired in the presence of IL-6.

Collectively, these data strongly indicate that IL-6 has a wide-ranging impact on the differentiation of CD4+ T cells both regarding their effector as well as their memory function.

Another finding of this study was that upon in vitro polarization, a larger proportion of CD4+ T cells lose expression of the costimulatory molecule CD28 among mTerc−/− G3 cells compared to controls.

Physiologically, CD28 expression on CD4+ T cells decreases transiently upon antigen recognition, but rapidly returns to normal levels [6]. However, repetitive stimulations as well as an increasing degree of differentiation generally decreases the expression of CD28 and loss of CD28 surface expression is deemed a characteristic of both cellular senescence and exhaustion of T cells [6, 47]. Importantly, loss of CD28 on T cell is a typical age-associated change of the immune system and leads to impaired responses towards antigen activation with age [3, 6]. In addition, signals via CD28 are required for the induction of telomerase, and downregulation of CD28 expression of inhibition of CD28-mediated signaling has been reported to lower telomerase activity [6, 15, 18]. Our new finding that telomerase-deficiency can cause CD28 downregulation in CD4+ T cells after stimulation indicates a reverse relationship or feedback loop, in which telomerase activity might be required to maintain CD28 expression.

Interestingly, CD28 also seems to play a central role in the regulation of IFNγ production, since several studies reported that CD4+ CD28- T cells secrete large amounts of IFNγ and have been associated with Th1-driven autoimmune diseases such as multiple sclerosis of rheumatoid arthritis [6, 48–50]. The loss of CD28 on stimulated mTerc−/− G3 CD4+ T cells might therefore be connected to the preference of telomerase-deficient cells to produce IFNγ, but this remains to be investigated in further studies.

In conclusion, we provide innovative data on the role of telomerase in CD4+ T cells, which demonstrate that the immunological phenotype of aged and telomerase-deficient mice shows striking similarities regarding the naïve T cell pool in steady-state as well as cytokine production and the expression of the costimulatory molecule CD28 after in vitro differentiation. Further, we identified IL-6 as an age-associated key molecule, which might influence the differentiation of telomerase-deficient Th1 and memory CD4+ T cells in vivo.

The findings of this study have to be seen in light of some limitations, as only generations G1 to G3 of mTerc−/− mice were investigated in this study. Furthermore, the effector function of CD4+ T cells was only assessed in vitro and CD8a + T cells were not examined in detail in this study. Therefore, research on later generations of telomerase-deficient mice as well as in vivo models will likely provide a more profound understanding of the effect of telomerase deficiency on both CD4+ as well as CD8a + T cells.

Still, the presented data strongly indicate that some of the changes observed in T cells of aged individuals could be associated with telomerase-associated mechanisms, which may be investigated in further studies. In addition to mice lacking the RNA component of telomerase (mTerc−/−), future research employing mice with a deficiency of the protein component Tert, which also has extratelomeric functions [51, 52], might improve even more insight into the cellular roles of telomerase.

## Methods

### Mice

Homozygous mTerc−/− and heterozygous mTerc +/− mice were kindly provided by Tobias Sperka (Leibniz Institute on Aging, Jena, Germany) and bred within our animal facility. mTerc +/+ control mice were offspring of mTerc +/− mice and were therefore littermates to the mTerc−/− G1 mice. All mice were between 6 and 16 weeks old. Both male and female animals were used, but no specific sex-related changes were observed. All experiments were performed in accordance to institutional guidelines and with protocols approved by the government of Middle Franconia.

### Harvest of lymphoid organs from mice

For isolation of mononuclear cells from blood, 200 μl of blood was taken from the facial vein of mice, mixed 1:1 with PBS and separated by density gradient centrifugation with *Ficoll® Paque Plus* (GE Healthcare). Bone marrow cells were obtained from the tibia by flushing the marrow content with PBS. Thymuses, mesenteric lymph nodes (mLNs) and spleens were dissociated into single cell suspensions between glass slides. To eliminate erythrocytes, splenocytes were treated with 2 ml ammonium-chloride-potassium lysis buffer for 3 min.

### Quantification of telomere length

Telomere length of cells in spleen and thymus was determined by a qPCR-based method using the *Absolute Mouse Telomere Length Quantification qPCR Assay Kit* by ScienCell according to the manufacturer’s recommendations.

### CD4+ T cell isolation and in vitro polarization

Naïve (CD25-) CD4+ T cells were isolated using the *CD4+ T Cell Isolation Kit, mouse* and the *CD25 MicroBead Kit, mouse* by Miltenyi Biotec. Instead of the recommended buffer in the kit, R10 medium (RPMI-1640 with 10% FCS (Gibco) and 1% Penicillin/Streptomycin (Sigma-Aldrich)) was used and the amount of reagents suggested per 10^7^ cells was instead used per 1.5 · 10^7^ cells.

For the polarization of the CD4+ CD25- T cells, a 24-well-plate was coated with 10 μg/ml anti-CD3 and 10 μg/ml anti-CD28 in PBS for 1 h at 37 °C and washed one time with PBS before the cells were seeded at a concentration of 10^6^ cells in R10 medium for Th0 and Th1 polarization. Lastly, 5 μg/ml anti-IL-4 (BioXcell) and 10 ng/ml IL-12 (R&D systems) diluted in PBS were added for Th1 polarization. For IL-6 treatment during polarization, 10 ng/ml or 100 ng/ml IL-6 (Miltenyi Biotec) were supplemented. The cells were in total cultured for five days at 37 °C and 5% CO_2_, with a medium change on day four. On day five, the cells were stimulated by addition of *eBioscience™ Cell Stimulation Cocktail (plus protein transport inhibitors) (500X)* (Invitrogen) for 3.5 h before harvest of supernatant for ELISA and cells for flow cytometry. The shown data after in vitro polarization are representative of at least four (Figs. 2, 3) or two (Fig. 4) independent experiments.

### Flow cytometry

Cells for flow cytometry staining were resuspended in PBS with 1% FCS treated with Fc block (CD16/CD32 Monoclonal Antibody (93) (Invitrogen)) for 10 min at 4 °C and stained with antibodies for cellular markers for 20 min at room temperature. For intracellular staining, cells were fixed and permeabilized using the *eBioscience™ Foxp3/Transcription Factor Staining Buffer Set* (Invitrogen) and stained for 20 min at room temperature. For the analysis of apoptosis, cells were resuspended in *eBioscience™ Binding Buffer for Annexin V* (Invitrogen) and stained with Annexin V (BioLegend) and propidium iodide (Invitrogen) for 15 min at room temperature. Flow cytometric measurements were performed at the MACSQuant Analyzer 10 (Miltenyi Biotec). Data analysis was performed with FlowJo™ software (FlowJo™ Software (for Windows) [software application] Version 10.6.2. Ashland, OR: Becton, Dickinson and Company; 2019.). Cells were gated for singlets, then lymphocytes, then CD4+ or CD8a + cells and subsequently the markers of interest.

The used antibodies included: CD4, T-bet, RORγT, FOXP3, IFNγ, CD27, CD44, CD62L, Ki-67 (Invitrogen); CD4, NRP1, CD28, Ki-67, CD49d (BioLegend); B220, GATA3 (Miltenyi Biotec); CD8a (BD Biosciences).

### Immunohistochemistry staining and quantification

Immunohistochemistry stainings were performed on paraffin-embedded spleens and mLNs from mice. Following deparaffinization, antigen retrieval and blocking, the slides were incubated with the primary antibody diluted 1:200 in TBST with 2% BSA overnight at 4 °C (*CD4 Monoclonal Antibody (4SM95), eBioscience™* (Invitrogen) or *Recombinant Anti-CD8 alpha antibody* (Abcam)) and with the secondary antibody diluted 1:1000 in TBS for 1 h at room temperature (*AffiniPure Goat Anti-Rabbit IgG (H + L)* (Jackson ImmunoResearch) or *Biotin Goat Anti-Rat Ig Clone Polyclonal (RUO) (554014*) (BD Biosciences)). A negative control was included by not adding first antibody on one tissue sample from each slide. Non-overlapping pictures (≥ 5 pictures per mLN (*n* ≥ 2) and ≥ 7 pictures per spleen (*n* = 3) per staining (CD4+ or CD8+)) of the whole organs were taken with the *Leica DMI4000 B inverted microscope* and analysed with ImageJ software (Rasband, W.S., ImageJ, U. S. National Institutes of Health, Bethesda, Maryland, USA, https://imagej.nih.gov/ij/, 1997–2018.). There, the two color channels were separately thresholded to generate a binary image, and the area fraction of the DAPI staining and the positive staining (CD4+ or CD8a+) were measured. Subsequently, the ratio between the CD4+ or CD8a + area relative to the area of DAPI staining was calculated.

## ELISA

For quantification of IFNγ and IL-6, the *ELISA MAX™ Standard Set Mouse IFN-γ* and *ELISA MAX™ Standard Set Mouse IL-6* (BioLegend) kits were used according to the manufacturer’s recommendations.

### Statistical analysis

A two-way ANOVA (analysis of variance) was performed using GraphPad Prism version 8 for Windows, GraphPad Software, La Jolla California USA, www.graphpad.com. Furthermore, the Dunnett’s multiple comparisons test (for three or more groups, i.e. mTerc +/+ vs. different generations of mTerc−/− mice) or the Šídák’s multiple comparisons test (for two groups, i.e. mTerc +/+ vs. mTerc−/− G3) was performed as post hoc test with a significance level of 5%.

## Supplementary Information

**Additional file 1: Supplementary Fig. 1.** Additional data on cell counts and composition of cell populations of mTerc−/− mice under steady-state conditions. **A** Total cell counts from organs obtained by counting of single cell suspensions using a hemocytometer (*n* = 3 for Terc+/+ and Terc−/− G2, *n* = 2 for Terc−/− G1). **B** Flow cytometric analysis of B cells (B220+, *n* = 3 from each generation) and NK cells (CD49d+, *n* = 3 for Terc+/+ and Terc−/− G2, *n* = 2 for Terc−/− G1) under steady-state conditions. **C** Analysis of markers of T helper cell subsets among CD4+ T cells from mTerc−/− mice under steady-state conditions (*n* = 3 from each generation). Graphs show the mean ± SD, * adjusted *p* ≤ 0.05, ** adjusted *p* ≤ 0.01, *** adjusted *p* ≤ 0.001. **Supplementary Fig. 2.** Additional data on CD4+ T cells from mTerc−/− mice under steady-state conditions. **A** Additional data on memory populations from lymphoid organs under steady-state conditions. Cells were defined as naïve (CD44- CD62L+), central memory (CD44+ CD62L+) and effector/effector memory (CD44+ CD62L-). **B** Proliferation of CD4+ T cells under steady-state conditions determined by Ki-67 expression in flow cytometry. **C** Cell death of CD4+ T cells under steady-state conditions as determined by flow cytometry. Cells were defined as live (Annexin V- PI-), early apoptotic (Annexin V+ PI-), late apoptotic (Annexin V+ PI+) and dead (Annexin V- PI+). The experiment was performed one time with *n* = 3 mice from each generation. Graphs show the mean ± SD, * adjusted *p* ≤ 0.05, ** adjusted p ≤ 0.01, *** adjusted p ≤ 0.001. **Supplementary Fig. 3. A and B** Further characterization of IFNγ secretion and proliferation of mTerc−/− CD4+ T cells during in vitro T cell polarization. **A** Daily quantification of IFNγ in the supernatant of the in vitro culture by ELISA. **B** and **C** Development of cell numbers during Th1 T cell polarization as determined by harvesting the cells and counting in a hemocytometer or quantification of total measured events in the lymphocyte gate in flow cytometry, respectively. The graphs show pooled data from *n* = 2 experiments, with a total number of *n* ≥ 6 technical replicates. Graphs show the mean ± SD, * adjusted p ≤ 0.05, ** adjusted *p* ≤ 0.01. **Supplementary Fig. 4.** Gating strategy for molecular markers assessed in flow cytometry. Representative gating strategy for flow cytometric analysis of memory populations, costimulatory molecules, proliferation and apoptosis after in vitro polarization.

## Data Availability

The datasets used and/or analysed during the current study are available from the corresponding author on reasonable request.
