# Supplementary material for: Telomerase deficiency reflects age-associated changes in CD4+ T cells
Source: Immun Ageing. 2022 Mar 23;19:16. doi: 10.1186/s12979-022-00273-0 (PMC8941756; doi:10.1186/s12979-022-00273-0)
Supplement: Supplementary file 1 — Additional file 1: Supplementary Fig. 1. Additional data on cell counts and composition of cell populations of mTerc−/− mice under steady-state conditions. A Total cell counts from organs obtained by counting of single cell suspensions using a hemocytometer (n = 3 for Terc+/+ and Terc−/− G2, n = 2 for Terc−/− G1). B Flow cytometric analysis of B cells (B220+, n = 3 from each generation) and NK cells (CD49d+, n = 3 for Terc+/+ and Terc−/− G2, n = 2 for Terc−/− G1) under steady-state conditions. C Analysis of markers of T helper cell subsets among CD4+ T cells from mTerc−/− mice under steady-state conditions (n = 3 from each generation). Graphs show the mean ± SD, * adjusted p ≤ 0.05, ** adjusted p ≤ 0.01, *** adjusted p ≤ 0.001. Supplementary Fig. 2. Additional data on CD4+ T cells from mTerc−/− mice under steady-state conditions. A Additional data on memory populations from lymphoid organs under steady-state conditions. Cells were defined as naïve (CD44- CD62L+), central memory (CD44+ CD62L+) and effector/effector memory (CD44+ CD62L-). B Proliferation of CD4+ T cells under steady-state conditions determined by Ki-67 expression in flow cytometry. C Cell death of CD4+ T cells under steady-state conditions as determined by flow cytometry. Cells were defined as live (Annexin V- PI-), early apoptotic (Annexin V+ PI-), late apoptotic (Annexin V+ PI+) and dead (Annexin V- PI+). The experiment was performed one time with n = 3 mice from each generation. Graphs show the mean ± SD, * adjusted p ≤ 0.05, ** adjusted p ≤ 0.01, *** adjusted p ≤ 0.001. Supplementary Fig. 3. A and B Further characterization of IFNγ secretion and proliferation of mTerc−/− CD4+ T cells during in vitro T cell polarization. A Daily quantification of IFNγ in the supernatant of the in vitro culture by ELISA. B and C Development of cell numbers during Th1 T cell polarization as determined by harvesting the cells and counting in a hemocytometer or quantification of total measured events in the lymphocyte [file 12979_2022_273_MOESM1_ESM.docx]

**Supplementary Material**

**
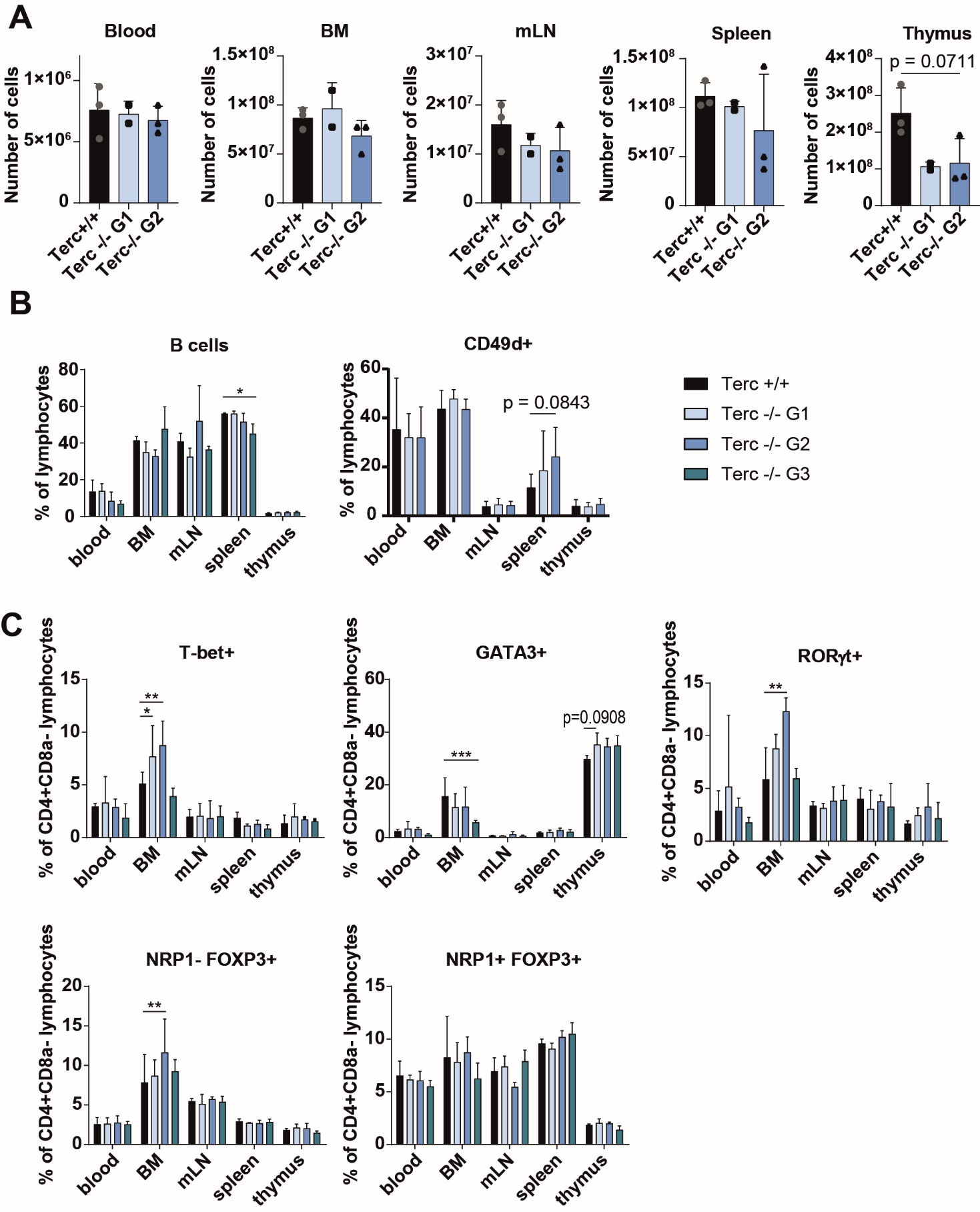
**

**Supplementary Figure 1*.* Additional data on cell counts and composition of cell populations of mTerc-/- mice under steady-state conditions.**

**(A)** Total cell counts from organs obtained by counting of single cell suspensions using a hemocytometer (n = 3 for Terc+/+ and Terc-/- G2, n = 2 for Terc-/- G1).

**(B)** Flow cytometric analysis of B cells (B220+, n = 3 from each generation) and NK cells (CD49d+, n = 3 for Terc+/+ and Terc-/- G2, n = 2 for Terc-/- G1) under steady-state conditions.

**(C)** Analysis of markers of T helper cell subsets among CD4+ T cells from mTerc-/- mice under steady-state conditions (n = 3 from each generation).

Graphs show the mean ± SD, * adjusted p ≤ 0.05, ** adjusted p ≤ 0.01, *** adjusted p ≤ 0.001.


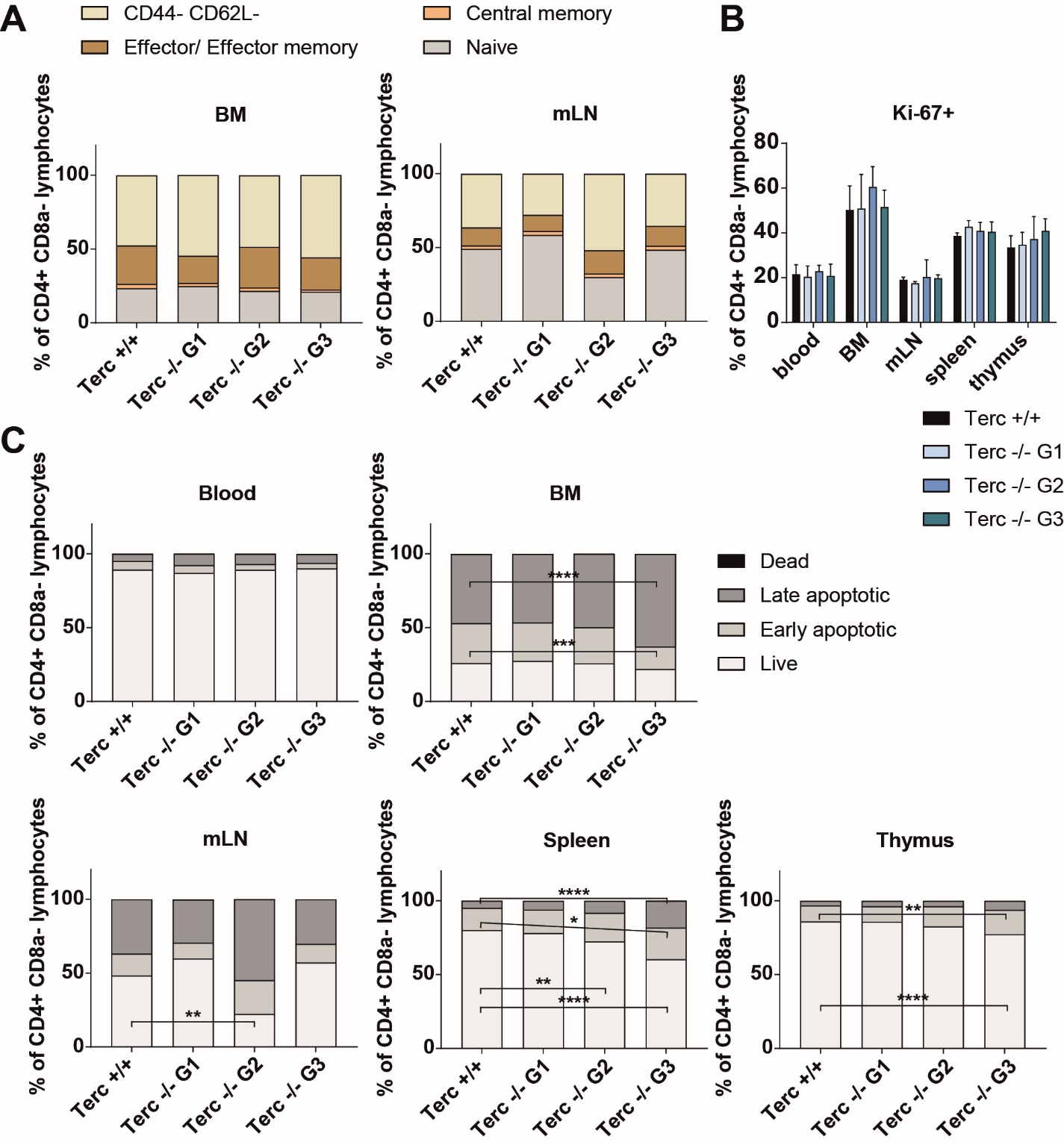


**Supplementary Figure 2. Additional data on CD4+ T cells from mTerc-/- mice under steady-state conditions.**

**(A)** Additional data on memory populations from lymphoid organs under steady-state conditions. Cells were defined as naïve (CD44- CD62L+), central memory (CD44+ CD62L+) and effector/effector memory (CD44+ CD62L-).

**(B)** Proliferation of CD4+ T cells under steady-state conditions determined by Ki-67 expression in flow cytometry.

**(C)** Cell death of CD4+ T cells under steady-state conditions as determined by flow cytometry. Cells were defined as live (Annexin V- PI-), early apoptotic (Annexin V+ PI-), late apoptotic (Annexin V+ PI+) and dead (Annexin V- PI+).

The experiment was performed one time with n = 3 mice from each generation. Graphs show the mean ± SD, * adjusted p ≤ 0.05, ** adjusted p ≤ 0.01, *** adjusted p ≤ 0.001.


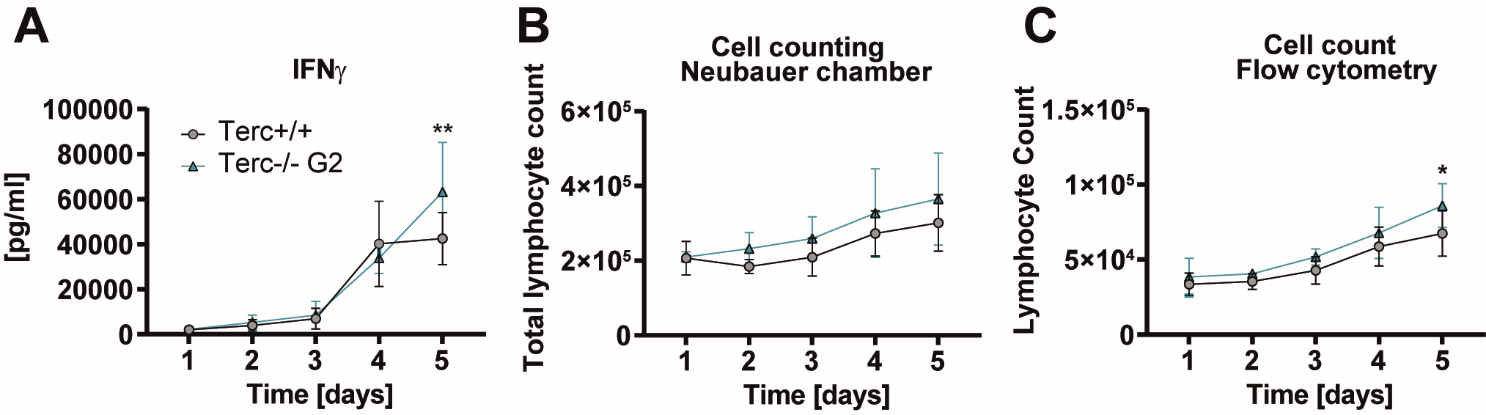


**Supplementary Figure 3. Further characterization of IFNγ secretion and proliferation of mTerc-/- CD4+ T cells during *in vitro* T cell polarization.**

**(A)** Daily quantification of IFNγ in the supernatant of the *in vitro* culture by ELISA.

**(B) and (C)** Development of cell numbers during Th1 T cell polarization as determined by harvesting the cells and counting in a hemocytometer or quantification of total measured events in the lymphocyte gate in flow cytometry, respectively.

The graphs show pooled data from n = 2 experiments, with a total number of n ≥ 6 technical replicates. Graphs show the mean ± SD, * adjusted p ≤ 0.05, ** adjusted p ≤ 0.01.


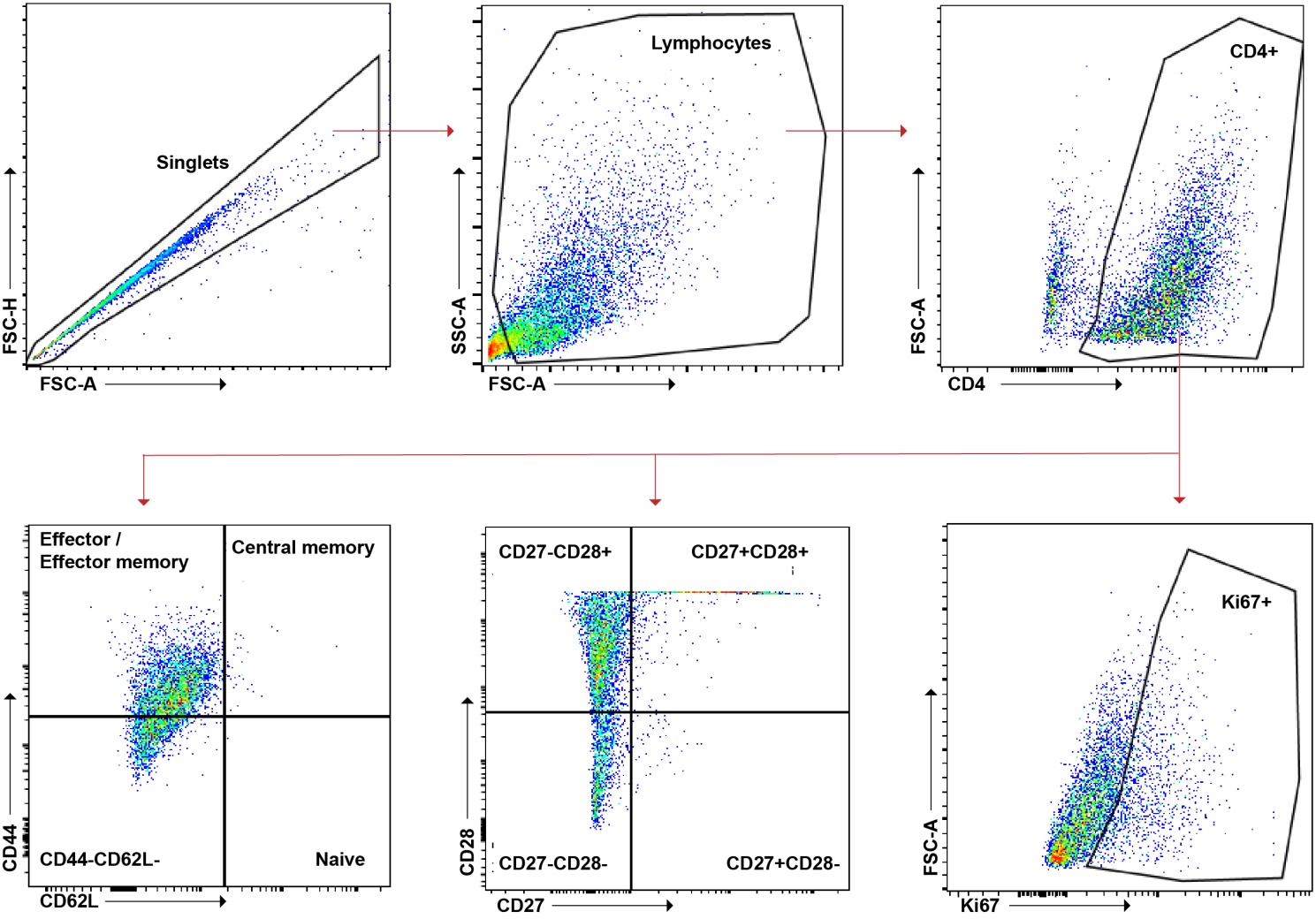


Supplementary Figure 4. Gating strategy for molecular markers assessed in flow cytometry.

Representative gating strategy for flow cytometric analysis of memory populations, costimulatory molecules, proliferation and apoptosis after *in vitro* polarization.
